# Ligand Free One-Pot Synthesis of Pyrano[2,3-*c*]pyrazoles in Water Extract of Banana Peel (WEB): A Green Chemistry Approach

**DOI:** 10.3389/fchem.2019.00944

**Published:** 2020-01-22

**Authors:** Kartikey Dhar Dwivedi, Biplob Borah, L. Raju Chowhan

**Affiliations:** Centre for Applied Chemistry, Central University of Gujarat, Gandhinagar, India

**Keywords:** pyrano[2,3-*c*]pyrazoles, web, arylidene malononitrile, 3-methyl-5-pyrazolone, green chemistry

## Abstract

Here, we have developed a novel, simple, efficient, and green protocol for one-pot synthesis of pyrano[2,3*-c*]pyrazole using arylidene malononitrile and pyrazolone in Water Extract of Banana Peels (WEB) as a reaction medium at room temperature (r.t.). This is a green and general synthetic protocol without utilization of any toxic organic solvent, ligand, base that could be applicable for the wide substrate scope in good to excellent yields. This protocol has various advantages such as fast reactions, eco-friendly reaction conditions, easy isolation of the product without using column chromatography. The green chemistry matrices calculation like atom economy reaction, environmental factor, as well as process mass intensity indicates the eco-friendly nature of the protocol.

## Introduction

The development of a reaction under green and mild condition by employing naturally available waste material is highly advantageous in organic synthesis (Marvaniya et al., 2011; Parmar et al., 2013; Maleki and Ashrafi, 2014). From the green chemistry point of view, the designing of a novel, efficient and clean reaction protocol is done by using a catalyst which is easy to separate, reusable and inexpensive. The synthesis of pharmaceutical product and fine chemicals via green chemistry approach has recently gained significant interest from academia and industry (Bazgir et al., 2013; Naeimi et al., 2014; Dalal et al., 2016; Dwivedi et al., 2018a,b, 2019). However, most of the organic synthesis uses catalysts which are expensive and toxic in nature. Therefore, choice of catalyst for green reaction is limited and has become a matter of concern for researchers nowadays (Kumarswamyreddy and Kesavan, 2016; Maddila et al., 2016b; Sebenzile et al., 2016; Zhang et al., 2017; Reddy et al., 2018). On the other hand, the use of renewable feedstock in organic transformations provides not only environmentally benign protocol but is also easily available in bulk (Saikia and Borah, 2015). The use of nature-derived reaction medium in organic synthesis successfully replaced toxic solvents, reagents, and expensive catalysts. Importantly, these methods are very efficient, suitable and generates negligible hazardous by-products (Saikia and Borah, 2015; Reddy et al., 2018). There are several naturally available plant based waste materials such as banana plants (trunk, rhizome, and peel) which have no use after collecting the banana fruits (Deka and Talukdar, 2007; Neog and Deka, 2013; Leitemberger et al., 2019). Finding a new method to use these type of waste material in organic transformation is worthful. Several organic transformations have been reported using WEB as reaction medium and catalyst such as synthesis of 3-carboxycoumarins (Bagul et al., 2017), Suzuki–Miyaura cross-coupling reactions (Boruah et al., 2015), The Henry reaction (Surneni et al., 2016) using WEB etc.

**Figure 1:**
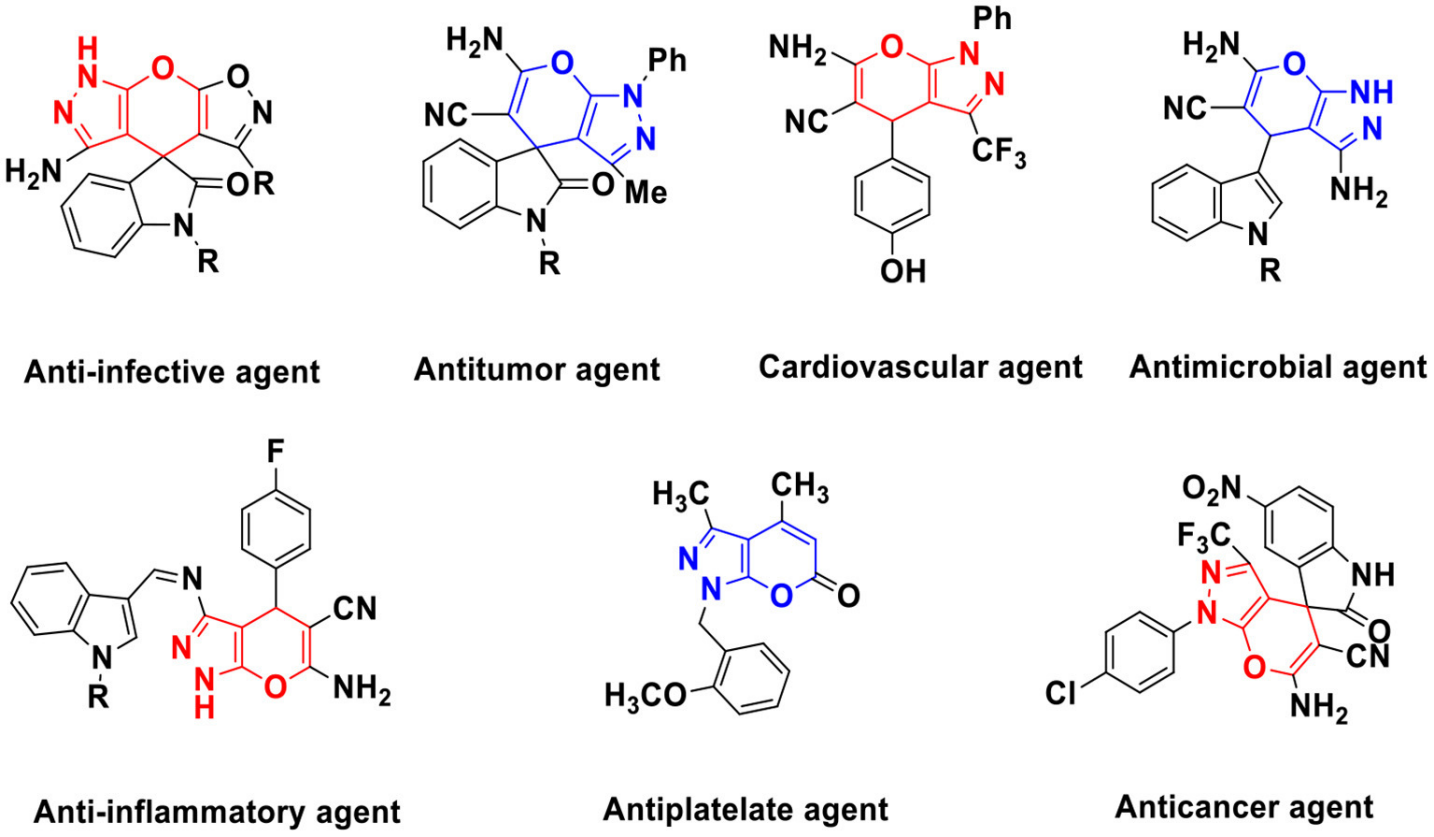
Some biologically active pyrano[2,3-*c*]pyrazole.

The pyranopyrazoles (Figure 1) is an important heterocyclic compound containing five-membered pyrazole ring fused with a six-membered pyran ring that occupies an important and wide area in medicinal chemistry (Maddila et al., 2017). Among other isomer the pyrano[2,3-*c*]pyrazole shows various biological activity including anti-bacterial (Das et al., 2014), anti-HIV (Fadda et al., 2013; Sirous et al., 2019), insecticidal, anti-tumors (Mariappan et al., 2010), antifungal, anti-cancer (Lalit et al., 2012) anti-inflammatory (Hamama et al., 2012), and analgesic (Mandour et al., 2012; Kasiotis et al., 2014). Owing to the biological importance, several methods for the synthesis of pyrano[2,3-*c*]pyrazole have been reported using different reaction condition like in EtOH (Junek and Aigner, 1973), piperidine (Vasuki and Kumaravel, 2008), morpholine (Sharanina et al., 1982), Et_3_N (Tacconi et al., 1980), L-Proline (Guo et al., 2007), neat (solvent free) (Nagarajan and Reddy, 2009), glycine (Reddy et al., 2010), nano-sized magnesium oxide (Babaie and Sheibani, 2011; Gangu et al., 2017), cupreine (Gogoi and Zhao, 2009), per-6-amino-β-cyclodextrin (per-6-ABCD catalyst) (Kanagaraj and Pitchumani, 2010). However, these methods have certain limitations viz. use of harsh reaction conditions, low yields, longer reaction time, tedious workup procedures, use of volatile and toxic organic solvent that negatively impact human health and the environment. Therefore, development of simple, clean, efficient, and high yielding protocol using natural waste materials and their extracts for the synthesis of pyrano[2,3-*c*]pyrazoles is highly desirable (Maddila et al., 2016a; Mamaghani and Hossein Nia, 2019; Shi et al., 2019). Therefore, here we describe an efficient ligand-free one-pot green methodology for the synthesis of pyranopyrazoles by the reaction of arylidene malononitrile and pyrazolone in WEB.

## Experimental Section

### General Experimental Detail

All commercially available chemicals were used without further purification. ^1^H NMR spectra were obtained on Bruker 500 MHz FT-NMR spectrometer. ^13^C NMR spectra were recorded at 125 MHz Chemical shifts are reported relative to the TMS signal. Multiplicity is indicated as follows: s (singlet); bs (broad singlet); d (doublet); t (triplet); q (quartet); m (multiplet); dd (doublet of doublets), etc. TOF and quadrupole mass analyzer types are used for the HRMS. FT-IR spectrometer (Shimadzu) in the range of 400–4,000 cm^−1^.

### General Procedure for the Synthesis of pyrano[2,3-*c*]pyrazole, 3(a-r)

To a solution of arylidene malononitrile (1 mmol) and WEB (3 ml/mmol), 3-methyl-5-pyrazolone (1 mmol) was added and the mixture was stirred for the indicated time (Table 1) at room temperature. The progresses of reaction was monitored by TLC (thin layer chromatography); however, the same can be inferred by disappearance of color and white precipitate formation. After completion of the reaction, as indicated by the TLC, the reaction mixture was filtered by using Whatman filter paper No 1 and washed with cold water. The obtained crude solid was then dissolved in ethyl acetate and passed through celite bed to remove any particulate impurities. The solvent was evaporated under reduced pressure and the obtained solid product was recrystallized by using methanol to give analytically pure 6-amino-3-methyl-4-phenyl-2,4-dihydro-pyrano[2,3-*c*]pyrazole-5-carbonitrile products (3a-3q) (Data shown in Supplementary Material).

**Table 1:** Optimization of reaction condition for the synthesis of pyrano[2,3-*c*]pyrazole^a^.

**Entry**	**Solvent**	**T^**°**^C**	**Time (min)^b^**	**Yield (%)^c^**
1	MeOH	r.t.	300	5%
2	EtOH	r.t.	300	5%
3	MeOH+ WEB(1:1)	r.t.	240	20
4	EtOH + WEB (1:1)	r.t.	120	25
5	MeOH +WEB(2:8)	r.t.	120	55
6	EtOH + WEB (2:8)	r.t.	120	60
7	DCM + WEB (1:1)	r.t.	240	25
8	DCM + WEB (2:8)	r.t.	120	40
9	WEB	r.t.	30	96

a *Reaction condition: All reactions were carried out on 1 mmol scale using equimolar amount of starting materials in WEB*.

b *Time for overall reaction*.

c *Isolated yield*.

### Spectral Data for Selected Compounds of pyrano[2,3-c]pyrazole (3a, 3d, 3l, 3o)

***6-amino-3-methyl-4-phenyl-2,4-dihydropyrano[2,3-c]pyrazole-5-carbonitrile, 3a:*** 89% yield, white solid. R_*f*_ = 0.45 (80% EtOAc/Hexane). M.P. 195–197°C IR (KBr) υ_*max*_ (cm^−1^) 3363, 3083, 2912, 1627, 1599, 1481, 1354, 1300, 1220, 1182, 1066, 867, 833, 761, 612; ^1^H NMR (500 MHz, CDCl_3_+DMSO-d_6_) δ 8.16 (s, 1H), 7.30 (t, *J* = 7.3 Hz, 2H), 7.21 (t, *J* = 6.3 Hz, 1H), 7.18 (t, *J* = 6.9 Hz, 2H), 6.71 (s, 2H), 4.55 (s, 1H), 1.80 (s, 3H). ^13^C NMR (125 MHz, CDCl_3_+DMSO-d_6_) δ 158.96, 152.91, 142.47, 133.59, 126.42, 125.56, 124.73, 118.88, 95.63, 55.40, 34.50, 7.87. HRMS (ESI^+^): m/z calculated for [C_14_H_12_N_4_O+H^+^]: 253.1089; found 253.1115.

***6-amino-4-(2-chlorophenyl)-3-methyl-2,4-dihydropyrano[2,3-c]pyrazole-5-carbonitrile, 3d:*** 92% yield, white solid. R_*f*_ = 0.40 (80% EtOAc/Hexane). M.P 214–215°C. IR (KBr) υ_*max*_ (cm^−1^) 3351, 3098, 2924, 1637, 1582, 1479, 1341, 1317, 1230, 1194, 1079, 878, 842, 751, 601; ^1^H NMR (500 MHz, CDCl_3_+DMSO-d_6_) δ 8.15 (s, 1H), 7.36 (d, *J* = 7.7 Hz, 1H), 7.28 (t, *J* = 7.2 Hz, 1H), 7.22 (t, *J* = 7.1 Hz, 1H), 7.17 (d, *J* = 7.1 Hz, 1H), 6.79 (s, 2H), 5.10 (s, 1H), 1.80 (s, 3H). ^13^C NMR (125 MHz, CDCl_3_+DMSO-d_6_) δ 158.93, 153.11, 145.59, 143.50, 133.84, 133.50, 119.20, 118.10, 113.51, 109.56, 95.97, 56.30, 53.85, 34.38, 8.18. HRMS (ESI+): m/z calculated for [C_14_H_11_ClN_4_O+H^+^]: 287.0700; found 287.0736.

***6-amino-4-(furan-2-yl)-3-methyl-2,4-dihydropyrano[2,3-c]pyrazole-5-carbonitrile, 3l:*** 91% yield, pale yellow color. R_*f*_ = 0.42 (80% EtOAc). M.P 215-217°C. IR (KBr) υ_*max*_ (cm^−1^) 3344, 3068, 2912, 1646, 1513, 1498, 1347, 1327, 1243, 1198, 1087, 875, 833, 751, 586; ^1^H NMR (500 MHz, CDCl_3_+DMSO-d_6_) δ 7.93 (s, 1H), 7.37 (s, 1H), 6.50 (s, 2H), 6.31 (s, 1H), 6.13 (s, 12H), 4.72 (s, 1H), 2.05 (s, 3H). ^13^C NMR (125 MHz, CDCl_3_+DMSO-d_6_) δ 160.10, 154.25, 140.16, 134.33, 119.15, 108.55, 103.95, 93.50, 52.89, 28.59, 8.23. HRMS (ESI+): m/z calculated for [C_12_H_10_N_4_O_2_+H^+^]: 243.0882; found 243.0921.

***6'-amino-1benzyl-3'-methyl-2-oxo-2'H-spiro[indoline-3,4'-pyrano[2,3-c]pyrazole]-5-carbonitrile, 3o:*** 87% yield, white solid. R_*f*_ = 0.49 (80% EtOAc/Hexane). M.P 236°C. IR (KBr) υ_*max*_ (cm^−1^) 3368, 3098, 2923, 1622, 1716, 1584, 1482, 1343, 1302, 1242, 1161, 1046, 852, 821, 752, 623; ^1^H NMR (500 MHz, CDCl_3_+DMSO-d_6_) δ 7.70 (s, *J* = 6.4 Hz, 1H), 7.39 (d, *J* = 7.2 Hz, 2H), 7.34–7.30 (m, 2H), 7.27 (d, *J* = 7.3 Hz, 1H), 7.22 (t, *J* = 7.5 Hz, 1H), 7.11 (d, *J* = 7.2 Hz, 1H), 7.07–7.03 (m, 1H), 6.84 (d, *J* = 7.7 Hz, 1H), 6.50 (s, 2H), 5.04 (d, *J* = 15.5 Hz, 1H), 4.88 (d, *J* = 15.5 Hz, 1H), 1.46 (s, *J* = 6.3 Hz, 3H). ^13^C NMR (125 MHz, CDCl_3_+DMSO-d_6_) δ 175.93, 161.88, 154.56, 140.97, 134.55, 134.25, 130.95, 127.90, 127.64, 126.60, 126.38, 123.64, 122.37, 117.82, 108.12, 94.21, 55.37, 46.20, 42.88, 8.36. HRMS (ESI^+^): m/z calculated for [C_22_H_17_N_5_O_2_+H^+^]: 384.1460; found 384.1506.

## Results and Discussion

### Preparation and Characterization of “Water Extract of Banana Peels” (WEB)

**Figure 2:**
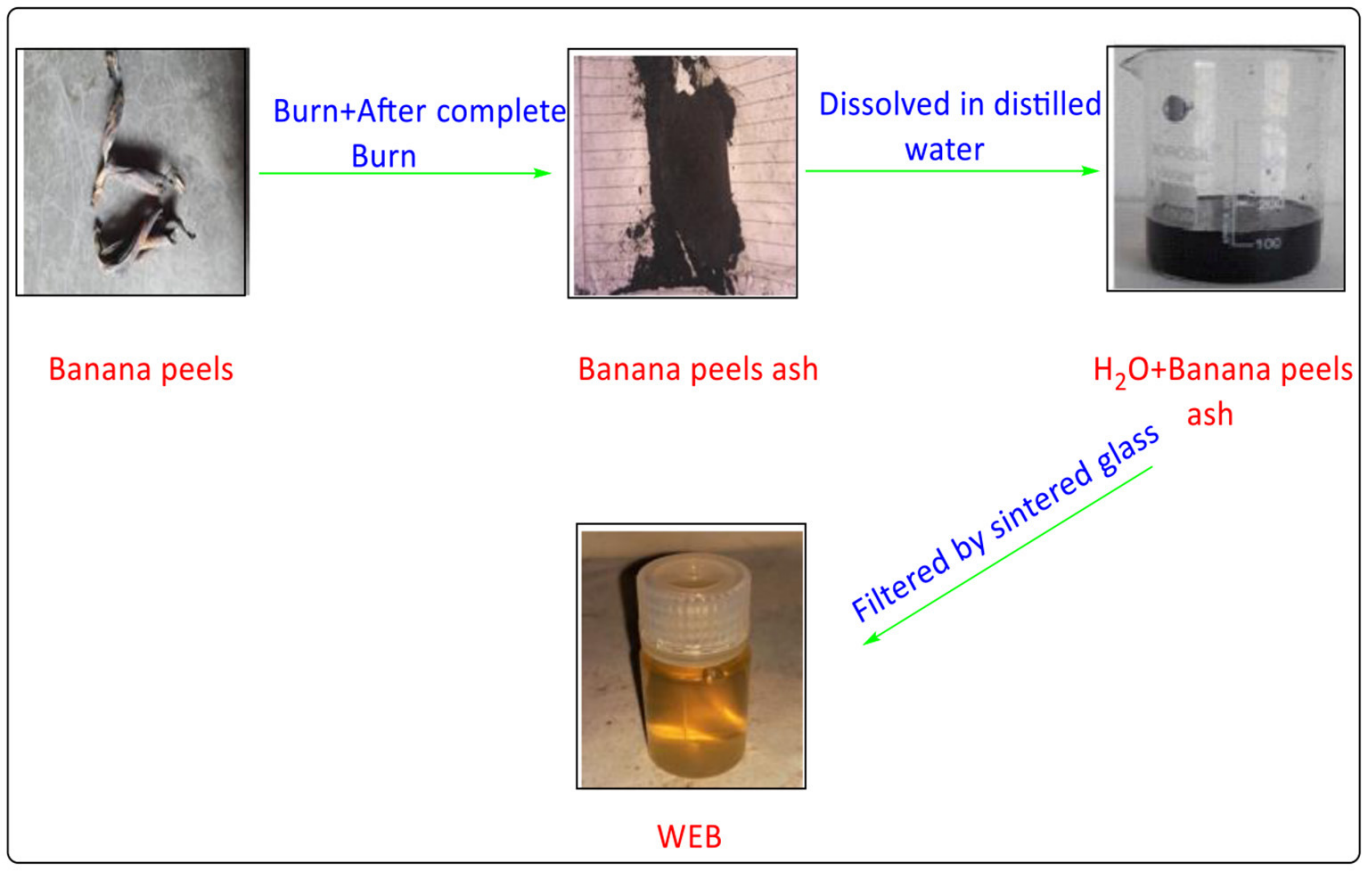
Preparation of water extract of banana peels (WEB).

Banana peels are naturally available waste material. The banana peels (Figure 2) was dried under sunlight and were burned to get the ash at 500°C for 2 h. This ash was transferred into a glass beaker containing distilled water (**3** gm of ash to **100** ml of distilled water) and the mixture was stirred for **10** min at room temperature. The slurry was filtered using a sintered glass funnel and the filtrate was termed as WEB (Boruah et al., 2015; Bagul et al., 2017). As mentioned earlier the WEB consists of different hydroxides and carbonated of metals such as Na and K etc. (Deka and Talukdar, 2007). These metal hydroxides and carbonates contributes the basicity of the extract. The pH of the extract was found to be **9.3**, which confirms the basic nature of the WEB and catalyzes the reaction.

**Scheme 1:**
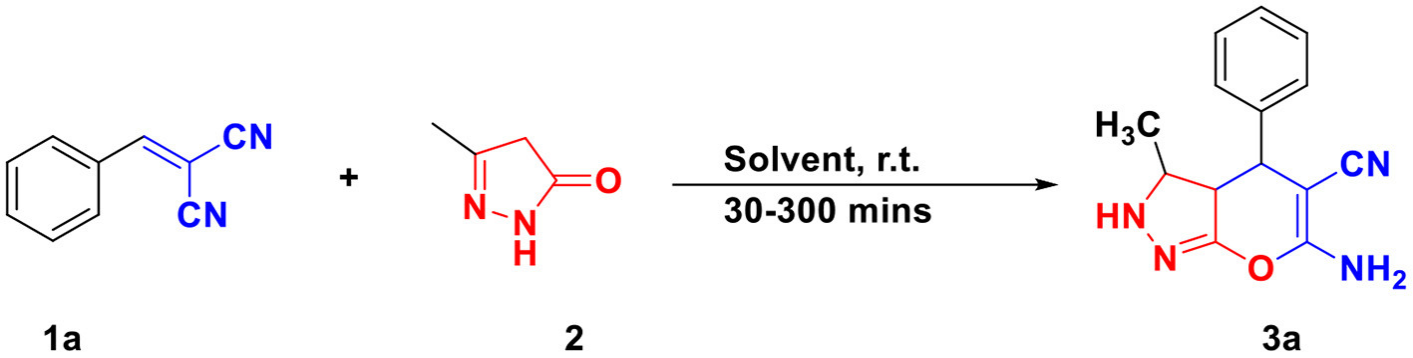
Optimization of reaction.

For optimizing the reaction condition, the model reaction was planned with an equimolar amount of arylidene malononitrile **1a** (1 mmol) and 3-methyl-5-pyrazolone **2** (1 mmol) in WEB under different solvents at room temperature (Scheme 1; Table 1). Initially, the reaction in simple methanol and ethanol as a solvent yielded trace amount of product even after 300 min (Table 1, entry 1,2). The reaction in a ratio of MeOH/EtOH to WEB in (1:1) afforded the desired product in 25% yield in 240 min (Table 1, entry 3,4). An increase in the ratio of MeOH:WEB (2:8) yielded the product in 55% (Table 1, entry 5,6). However, the yield was decreased when the reaction was performed in combination of DCM and WEB (Table 1, entry 7, 8). Thereafter, the reaction was examined in neat WEB which afforded the desired product in 96% (Table 1, Entries 9). Thus, by using WEB the yield of product was excellent and found to be the best optimized condition for the synthesis of pyrano[2,3-*c*]pyrazole as reaction medium without using other organic solvents.

**Scheme 2:**
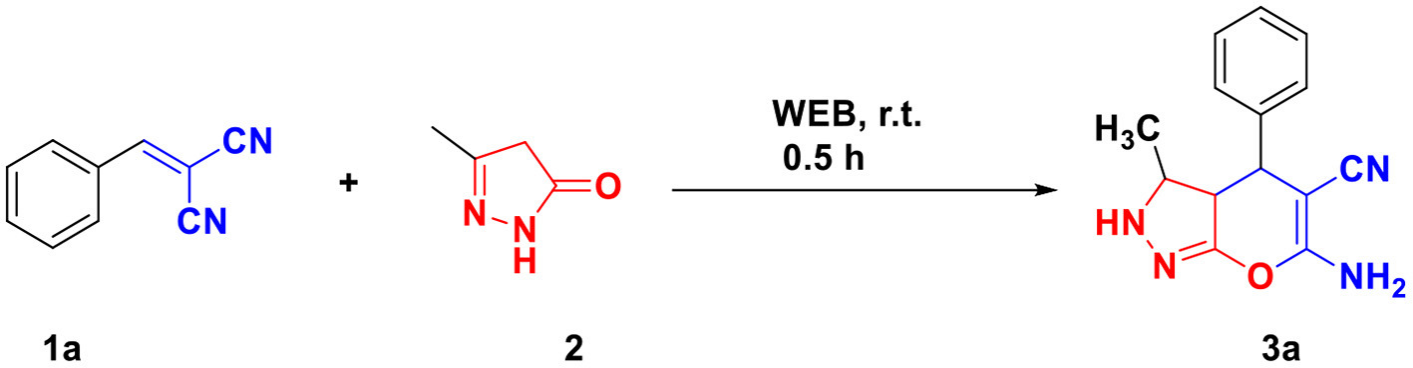
Optimization of quantity of WEB.

To optimize the amount of WEB required for the reaction, the reaction was performed in different amount of WEB and equimolar amount of arylidene malononitrile **1a** (1 mmol), 3-methyl-5-pyrazolone **2** (1 mmol) at room temperature. It was found that 3 ml of WEB is sufficient enough to complete the reaction in 30 min to give quantitative amount of product (Scheme 2).

By employing the optimized condition, the reaction was performed with various substituted arylidene malononitrile 1a-q and 3-methyl-5-pyrazolone 2 in WEB at r.t. The methodology was found to have wide substrate scope. All halogenated substrates afforded the pyrano[2,3-*c*]pyrazoles 3 with excellent yield without any side reaction. It is interesting to note that different arylidene malononitrile with various electron-withdrawing group and electron-donating groups at C-2, C-3, C-4, position successfully give the desired product in quantitative yield (Table 2).

**Table 2:** Substrate scope for the synthesis of pyrano[2,3-*c*]pyrazoles.

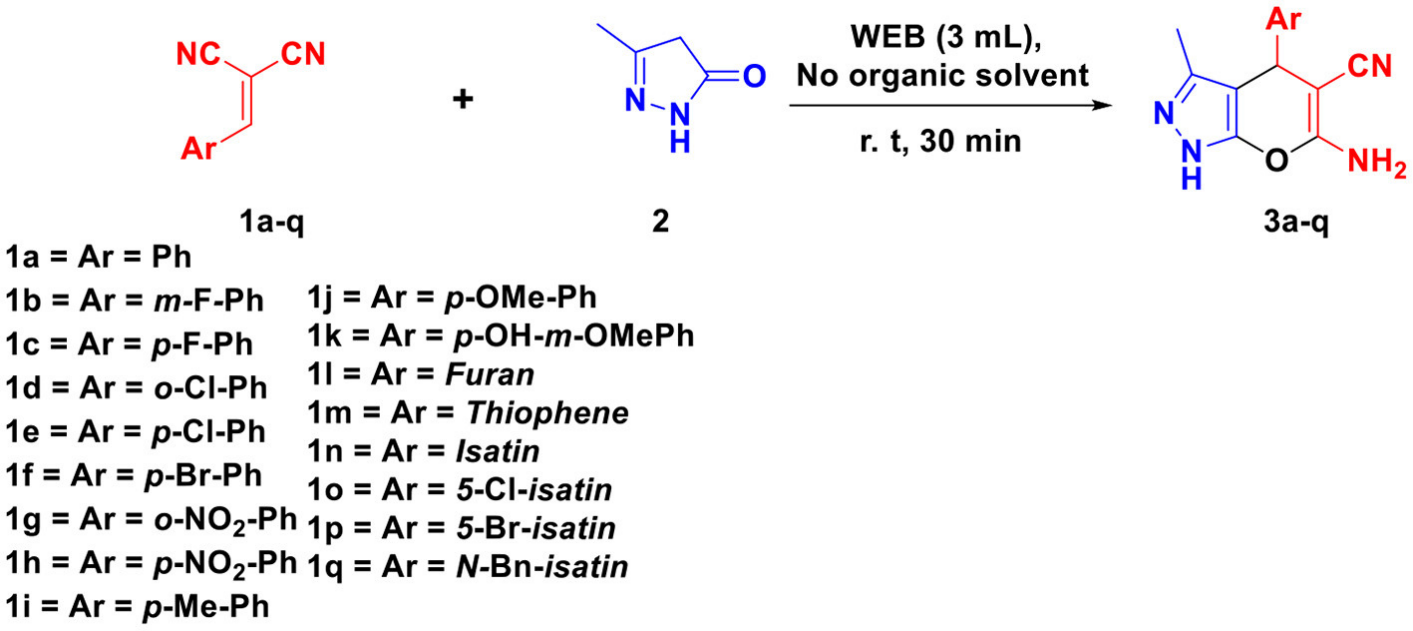
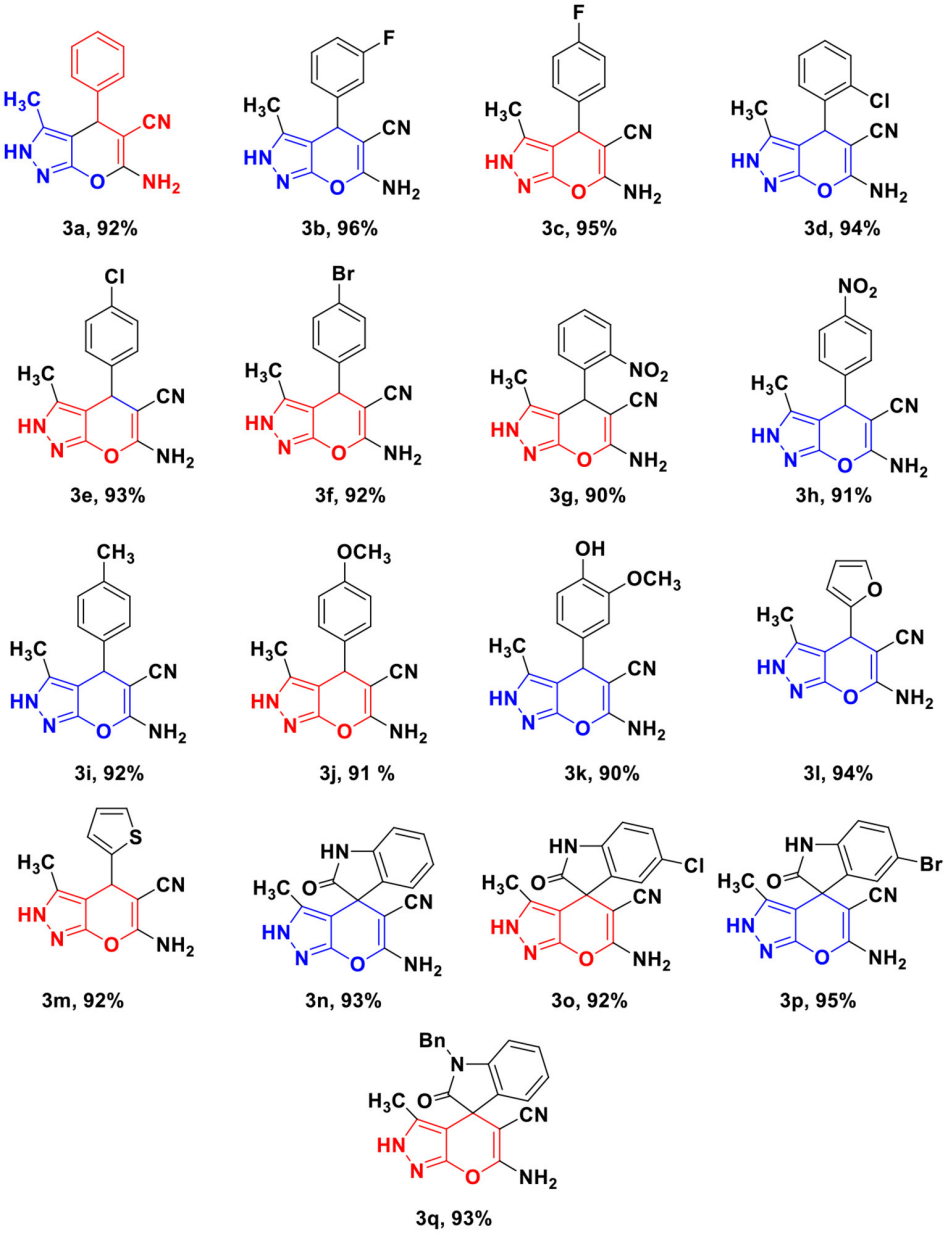

**Scheme 3:**
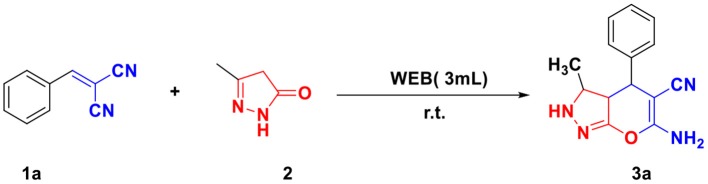
Scale-up experiment.

To further explore the scale-up performance of this protocol for one-pot preparation of pyrano[2,3-*c*]pyrazole derivative **3a** which is important for possible large-scale application. Therefore, we performed a gram-scale reaction for the preparation of **3a** (10 mmol) and the yield obtained was quantitative in nature (Scheme 3).

Green chemistry matrices (Bahuguna et al., 2017; Chowhan et al., 2017) like atom economy (A.E.) reaction mass efficiency (R. M. E) should be high, and environmental factor (E), as well as process mass intensity (P. M. I) should be low (Constable et al., 2002). Green chemistry matrixes were calculated for the reaction, and we found low E-factor (0.086), P. M. I (1.164), high R. M. E (91.99%), high atom economy (A.E. = 100%), and process mass intensity (P.M.I. factor = 1.164). These values clearly indicate the efficacy of the present protocol.

**Figure 3:**
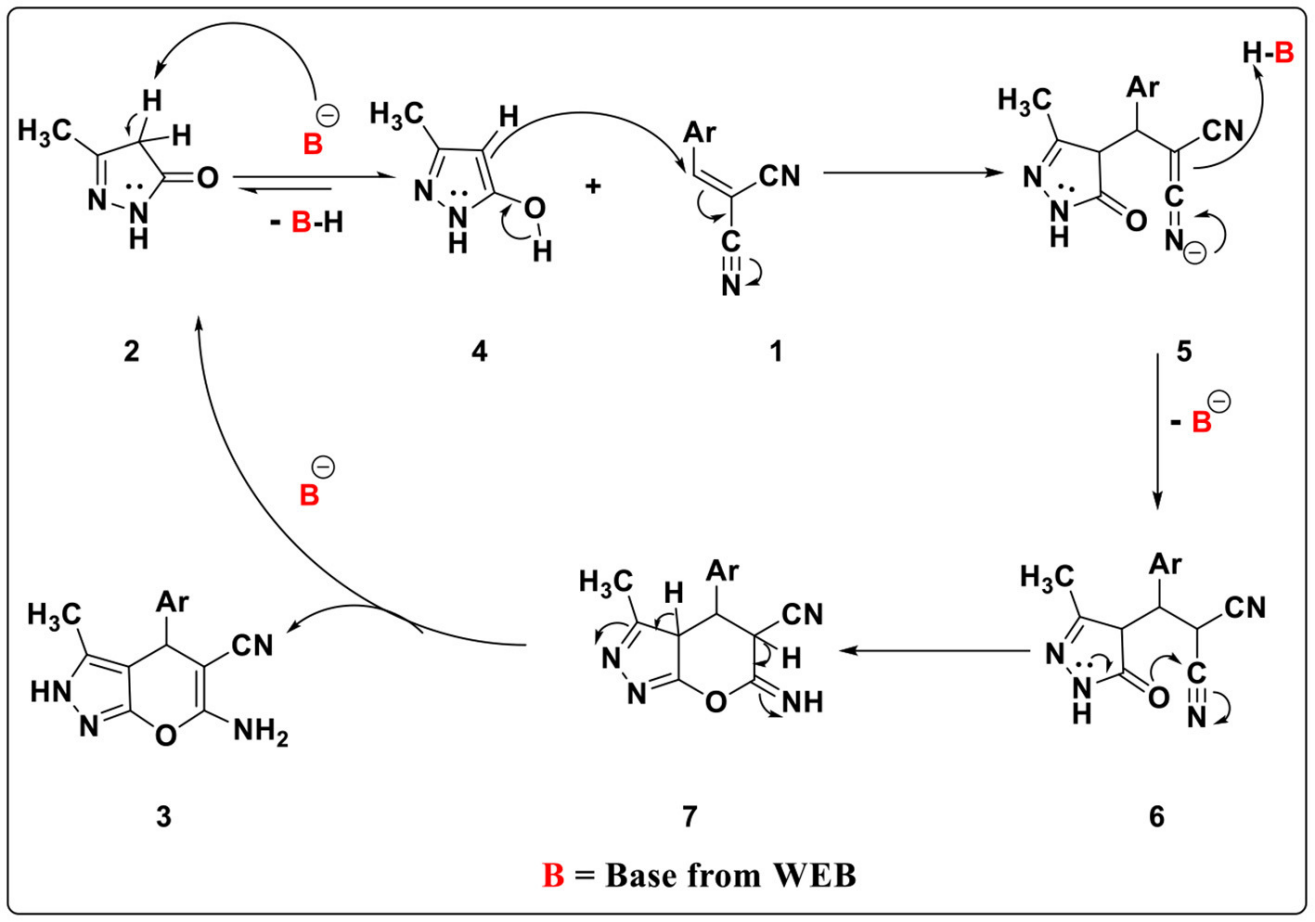
Plausible mechanism for the synthesis of pyrano[2,3-*c*]pyrazole by using WEB.

The mechanism involved is as follows. Initially, in the presence of WEB, 3-methyl-5-pyrazolone **2** can form its enolic form **4**, which undergoes Michael addition with arylidene malononitrile **1** to give intermediate **5** (Figure 3). Abstraction of the proton from B-H by intermediate **5** generates the intermediate **6** which could undergo intramolecular cyclization and give the intermediate **7** which could isomerizes and gives the desired product **3** (Ahadi et al., 2010; Zou et al., 2011).

## Conclusions

A simple, efficient and green protocol has been developed for the synthesis of pyrano[2,3-*c*]pyrazole at room temperature using WEB as reaction medium without using other organic solvents, base, additives. The method has a broad range of substrate scope. The waste material was successfully used as a reaction medium for the organic transformation. The main advantage of this method is mild reaction condition, faster reaction, high yield, ecofriendly, and sustainable from the economic point of view. Calculated green chemistry matrices calculated prove the efficacy of the protocol. The method is very efficient for practical synthesis. Therefore, waste derived reaction medium can be efficient and ecofriendly alternative for the organic synthesis.

## Data Availability Statement

All datasets generated for this study are included in the article/Supplementary Material.

## Author Contributions

KD, BB, and LC contributed in designing the work, execution, and analysis of the results.

### Conflict of Interest

The authors declare that the research was conducted in the absence of any commercial or financial relationships that could be construed as a potential conflict of interest.
