# Supplementary material for: Ligand Free One-Pot Synthesis of Pyrano[2,3-c]pyrazoles in Water Extract of Banana Peel (WEB): A Green Chemistry Approach
Source: Front Chem. 2020 Jan 22;7:944. doi: 10.3389/fchem.2019.00944 (PMC6987396; doi:10.3389/fchem.2019.00944)
Supplement: Supplementary file 1 [file Table_1.docx]

***Supplementary Material (ESI)***

Ligand Free One-Pot Synthesis of Pyrano[2,3-c]pyrazoles in Water Extract of Banana Peel (WEB): A Green Chemistry Approach

Kartikey Dhar Dwivedi, Biplob Borah and L. Raju Chowhan*^a^

**Table of contents**

| S. No. | Section | Page No. |
| --- | --- | --- |
| 1. | General | S2 |
| 2. | General experimental procedure for the reaction | S2 |
| 3. | Green matrices calculation for the reactions | S2-S3 |
| 4. | Spectral data for products (3a-3q) | S4-S11 |
| 5. | Copies of ^1^H and ^13^C NMR spectra of products | S12-S28 |

**General**

All commercially available chemicals were used without further purification. ^1^H NMR spectra were obtained on Bruker 500 MHz FT-NMR spectrometers. ^13^C NMR spectra were recorded at 125 MHz. Chemical shifts are reported in relative to the TMS signal. Multiplicity is indicated as follows: s (singlet); bs (broad singlet); d (doublet); t (triplet); q (quartet); m (multiplet); dd (doublet of doublets), etc. TOF and quadrupole mass analyzer types are used for the HRMS measurements IR spectra were recorded on FT-IR-5700 instrument.

**General experimental procedure for the reaction**

To a solution of arylidene malononitrile (1 mmol) and WEB (3ml), 3-methyl-5-pyrazolone (1 mmol) were added the mixture was stirred for the indicated time (**Table 1**) at room temperature. The reaction progresses were determined by TLC (thin layer chromatography), however, the same can be inferred by visualising the disappearance of arylidene malononitrile colour and white solid formation. After the consumption of starting material, as indicated by the TLC, the reaction mixture was filtered by using whatman filter paper No1 and washed with cold water (4 mL). The obtained crude solid was then dissolved in ethyl acetate (5 mL) and passed through celite bed to remove any particulate impurities from the product, concentrated under reduced pressure. Crude was recrystallized from methanol to give analytically pure 6-amino-3-methyl-4-phenyl-2,4-dihydropyrano[2,3-c]pyrazole-5-carbonitrile products **(3a-3q)**.

1. **Green matrices calculation for the reaction**

***Atom economy (AE):*** AE of a chemical reaction defines how many atoms from the starting materials reside within the product. It is considered a measure of the efficiency of a reaction.

The ideal value of AE factor is 100% (which means all atoms from the starting materials still reside in the product).

*AE* = Mol. Wt. of product ÷ Σ(MW of stoichiometric reactants) × 100

*AE* = 252.277 g/mol ÷ (154.172 g/mol + 98.105 g/mol) × 100

*AE* = 100%.

***E-factor or environmental factor E-factor****:* The E-factor is a popular Green chemistry matrix

Which is defined by the ratio of the mass of waste per mass of the product.

*E-factor* = [mass of waste]/ mass of product

Mass of waste = total mass of raw materials - Total mass of product

So,

*E- Factor* = (154.172 + 98.105) – 232.09 mg/ 232.09 mg

*E- Factor* = 0.086(Ideal value of E-factor is considered zero)

***Reaction mass efficiency (RME):*** Reaction mass efficiency is defined as the mass of product divided by the sum of the total mass of stoichiometric reactants. Value of RME ranges from 0-100 %. The larger number is considered better and it measures the “cleanness” of a chemical reaction.

*RME* = mass of product / Σ(mass of stoichiometric reactants) × 100

*RME* = 232.09 ÷ (154.172 + 98.105) × 100

= 91.99%

***Product mass intensity (PMI)*:** PMI is the total mass used in a chemical process divided by the mass of product. In PMI solvent is also considered, hence water also adds value to PMI.

*PMI* = Σ(mass of stoichiometric reactants + solvent) / mass of product

*PMI* = (154.172 + 98.105 + 18.01 mg) ÷ 232.09 mg

= 1.164

| ***Compound*** | ***Atom economy (AE) (***%) | ***E-factor*** | ***Reaction mass efficiency (RME)*** | ***Product mass intensity (PMI)*** |
| --- | --- | --- | --- | --- |
| *3a* | 100% | 0.086 | 91.99% | 1.164 |

**6-amino-3-methyl-4-phenyl-2,4-dihydropyrano[2,3-*c*]pyrazole-5-carbonitrile, 3a:**

89% yield, white solid. R_f_ = 0.45 (80% EtOAc/Hexane). M.P. 195-197°C IR (KBr) *υ_max_* (cm^-1^) 3363, 3083, 2912, 1627, 1599, 1481, 1354, 1300, 1220, 1182, 1066, 867, 833, 761, 612; ^1^H NMR (500 MHz, CDCl_3_) δ 8.16 (s, 1H), 7.30 (t, *J* = 7.3 Hz, 2H), 7.21 (t, *J* = 6.3 Hz, 1H), 7.18 (t, *J* = 6.9 Hz, 2H), 6.71 (s, 2H), 4.55 (s, 1H), 1.80 (s, 3H). ^13^C NMR (126 MHz, CDCl_3_) δ 158.96, 152.91, 142.47, 133.59, 126.42, 125.56, 124.73, 118.88, 95.63, 55.40, 34.50, 7.87. HRMS (ESI^+^): m/z calculated for [C_14_H_12_N_4_O+H^+^]: 253.1089; found 253.1115.

**6-amino-4-(3-flurophenyl)-3-methyl-2,4-dihydropyrano[2,3*-c*]pyrazole-5-carbonitrile,3b :** 96% yield, white solid. R_f_ = 0.40 (80% EtOAc/Hexane). M.P 228-230°C. IR (KBr) *υ_max_* (cm^-1^) 3362, 3084, 2913, 1626, 1591, 1488, 1352, 1303, 1221, 1185, 1068, 865, 830, 762, 611; ^1^H NMR (500 MHz, CDCl_3_) δ 7.33 (dd, *J* = 14.0, 7.6 Hz, 1H), 7.03 (d, *J* = 7.5 Hz, 1H), 6.98 (t, *J* = 7.7 Hz, 1H), 6.92 (d, *J* = 9.7 Hz, 1H), 6.79 (s, 2H), 4.61 (s, 1H), 1.84 (s, 3H). ^13^C NMR (126 MHz, CDCl_3_) δ 161.60, 159.65, 159.27, 153.09, 145.51, 145.46, 133.94, 128.39, 128.32, 121.71, 118.96, 112.49, 112.32, 111.89, 111.73, 95.18, 55.19, 34.48, 8.11. HRMS(ESI+): m/z calculated for [C_14_H_11_FN_4_O+H^+^]: 271.0995; found 271.0965

**6-amino-4-(4-flurophenyl)-3-methyl-2,4-dihydropyrano[2,3-*c*]pyrazole-5-carbonitrile, 3c:** 94% yield, white solid. R_f_ = 0.40 (80% EtOAc/Hexane). M.P 202-203°C. IR (KBr) *υ_max_* (cm^-1^) 3364, 3085, 2914, 1627, 1592, 1489, 1351, 1307, 1220, 1184, 1069, 868, 832, 761, 610; ^1^H NMR (500 MHz, CDCl_3_) δ 7.22 – 7.16 (m, 2H), 7.01 (t, *J* = 8.4 Hz, 2H), 6.39 (s, 2H), 4.57 (s, 1H), 1.82 (s, 3H). ^13^C NMR (126 MHz, CDCl_3_) δ 160.95, 159.54, 159.01, 153.70, 138.66, 138.64, 134.56, 127.95, 127.88, 119.52, 113.83, 113.66, 95.99, 56.91, 34.68, 8.68. HRMS (ESI+): m/z calculated for [C_14_H_11_FN_4_O+H^+^]: 271.0995; found 271.1035.

**6-amino-4-(2-chlorophenyl)-3-methyl-2,4-dihydropyrano[2,3-*c*]pyrazole-5-carbonitrile, 3d:** 92% yield, white solid. R_f_ = 0.40 (80% EtOAc/Hexane). M.P 214-215°C. IR (KBr) *υ_max_* (cm^-1^) 3351, 3098, 2924, 1637, 1582, 1479, 1341, 1317, 1230, 1194, 1079, 878, 842, 751, 601; ^1^H NMR (500 MHz, CDCl_3_) δ 8.15 (s, 1H), 7.36 (d, *J* = 7.7 Hz, 1H), 7.28 (t, *J* = 7.2 Hz, 1H), 7.22 (t, *J* = 7.1 Hz, 1H), 7.17 (d, *J* = 7.1 Hz, 1H), 6.79 (s, 2H), 5.10 (s, 1H), 1.80 (s, 3H). ^13^C NMR (126 MHz, DMSO) δ 158.93, 153.11, 145.59, 143.50, 133.84, 133.50, 119.20, 118.10, 113.51, 109.56, 95.97, 56.30, 53.85, 34.38, 8.18. HRMS (ESI+): m/z calculated for [C_14_H_11_ClN_4_O+H^+^]: 287.0700; found 287.0736.

**6-amino-4-(4-chlorophenyl)-3-methyl-2,4-dihydropyrano[2,3-*c*]pyrazole-5-carbonitrile, 3e:** 93% yield, white solid. R_f_ = 0.40 (80% EtOAc/Hexane). M.P 207-209°C. IR (KBr) *υ_max_* (cm^-1^) 3355, 3097, 2923, 1636, 1583, 1478, 1342, 1316, 1231, 1195, 1089, 888, 832, 753, 593; ^1^H NMR (500 MHz, CDCl_3_) δ 7.41 (s, 1H), 7.27 (d, *J* = 7.4 Hz, 2H), 7.15 (d, *J* = 7.9 Hz, 2H), 5.50 (s, 2H), 4.58 (s, 1H), 1.85 (s, 3H). ^13^C NMR (126 MHz, CDCl_3_) δ 159.63, 153.68, 141.45, 134.59, 130.65, 127.84, 127.08, 119.45, 95.70, 56.55, 34.81, 8.70. HRMS (ESI+): m/z calculated for [C_14_H_11_ClN_4_O+H^+^]: 287.0700; found 287.0721

**6-amino-4-(4-bromophenyl)-3-methyl-2,4-dihydropyrano[2,3-*c*]pyrazole-5-carbonitrile, 3f:** 92% yield, white solid. R_f_ = 0.40 (80% EtOAc/Hexane). M.P 220-221°C. IR (KBr) *υ_max_* (cm^-1^) 3355, 3097, 2923, 1636, 1583, 1478, 1342, 1316, 1231, 1195, 1089, 888, 832, 741, 590; ^1^H NMR (500 MHz, CDCl_3_) δ 8.06 (s, 1H), 7.44 (d, *J* = 8.1 Hz, 2H), 7.12 (d, *J* = 8.0 Hz, 2H), 6.66 (s, 2H), 4.56 (s, 1H), 1.82 (s, 3H). ^13^C NMR (126 MHz, CDCl_3_) δ 159.39, 153.34, 141.89, 134.18, 129.70, 127.99, 119.16, 118.52, 95.36, 55.76, 34.56, 8.39. HRMS(ESI+): m/z calculated for [C_14_H_11_BrN_4_O+H^+^]: 331.0194; found 331.0229.

**6-amino-4-(2-nitrophenyl)-3-methyl-2,4-dihydropyrano[2,3-*c*]pyrazole-5-carbonitrile, 3g:** 80% yield, white solid. R_f_ = 0.40 (80% EtOAc/Hexane). M.P 221-223°C. IR (KBr) *υ_max_* (cm^-1^) 3355, 3097, 2923, 1636, 1518, 1468, 1348, 1317, 1233, 1197, 1087, 887, 833, 752, 596;  ^1^H NMR (500 MHz, CDCl_3_) δ 12.24 (s, 1H), 8.73 (s, 1H), 8.15 (s, 1H), 7.56 (s, 1H), 7.52 (s, 2H), 4.04 (s, 1H), 2.60 (s, 3H). ^13^C NMR (126 MHz, CDCl_3_) δ 163.42, 145.03, 138.80, 131.42, 129.22, 126.72, 124.88, 124.61, 120.97, 115.48, 106.39, 51.22, 20.69. HRMS (ESI+): m/z calculated for [C_14_H_11_N_5_O_3_+H^+^]: 298.0940; found 298.0805.

**6-amino-4-(4-nitrophenyl)-3-methyl-2,4-dihydropyrano[2,3-*c*]pyrazole-5-carbonitrile, 3h:** 88% yield, white solid. R_f_ = 0.42 (80% EtOAc/Hexane). M.P 209-210°C. IR (KBr) *υ_max_* (cm^-1^) 3345, 3067, 2913, 1656, 1517, 1478, 1347, 1327, 1243, 1198, 1088, 871, 843, 754, 596; ^1^H NMR (500 MHz, CDCl_3_) δ 8.18 (t, *J* = 6.3 Hz, 2H), 7.52 (d, *J* = 4.8 Hz, 1H), 7.42 (d, *J* = 6.2 Hz, 2H), 5.94 (s, *J* = 4.2 Hz, 2H), 4.74 (s, *J* = 4.8 Hz, 1H), 1.86 (s, *J* = 4.8 Hz, 3H). ^13^C NMR (126 MHz, CDCl_3_) δ 159.85, 153.60, 150.23, 145.21, 134.56, 127.30, 122.32, 119.15, 94.92, 55.38, 35.17, 8.62. HRMS (ESI^+^): m/z calculated for [C_14_H_11_N_5_O_3_+H^+^]: 298.0940; found 298.0809

**6-amino-4-(p-tolyl)-3-methyl-2,4-dihydropyrano[2,3-*c*]pyrazole-5-carbonitrile, 3i:**

87% yield, white solid. R_f_ = 0.45 (80% EtOAc/Hexane). M.P 230-232°C. IR (KBr) *υ_max_* (cm^-1^) 3344, 3068, 2912, 1646, 1513, 1498, 1347, 1327, 1243, 1198, 1087, 875, 833, 751, 586; ^1^H NMR (500 MHz, CDCl_3_) δ 7.47 (s, 1H), 7.09 (s, 4H), 5.51 (s, 2H), 4.54 (s, 1H), 2.31 (s, 3H), 1.85 (s, 3H). ^13^C NMR (126 MHz, CDCl_3_) δ 159.16, 153.27, 139.56, 134.11, 133.91, 127.25, 125.73, 119.26, 95.94, 56.23, 34.54, 19.15, 8.25. HRMS (ESI+): m/z calculated for [C_15_H_14_N_4_O+H^+^]: 267.1246; found 267.1112

**6-amino-4-(4-methoxyphenyl)-3-methyl-2,4-dihydropyrano[2,3-*c*]pyrazole-5-carbonitrile, 3j:** 85% yield, white solid. R_f_ = 0.50 (80% EtOAc/Hexane). M.P 235-237°C. IR (KBr) *υ_max_* (cm^-1^) 3340, 3078, 2932, 1636, 1512, 1491, 1348, 1326, 1242, 1197, 1086, 874, 823, 758, 580; ^1^H NMR (500 MHz, CDCl_3_) δ 7.43 (s, *J* = 2.1 Hz, 1H), 7.11 (d, *J* = 6.0 Hz, 2H), 6.82 (d, *J* = 6.1 Hz, 2H), 5.42 (s, 2H), 4.54 (s, 1H), 3.79 (s, *J* = 2.2 Hz, 3H), 1.84 (s, *J* = 1.8 Hz, 3H). ^13^C NMR (126 MHz, CDCl_3_) δ 158.95, 156.31, 153.16, 134.38, 134.00, 126.76, 119.21, 111.82, 95.95, 56.57, 53.17, 34.04, 8.16. HRMS (ESI+): m/z calculated for [C_15_H_14_N_4_O_2_+H^+^]: 283.1195; found 283.1216.

**6-amino-4-(4-hydroxy-3-methoxyphenyl)-3-methyl-2,4-dihydropyrano[2,3-*c*]pyrazole-5-carbonitrile, 3k:** 84% yield, white solid. R_f_ = 0.40 (80% EtOAc/Hexane). M.P 239-241°C. IR (KBr) *υ_max_* (cm^-1^) 3340, 3078, 2932, 1636, 1512, 1491, 1348, 1326, 1242, 1197, 1086, 874, 823, 758, 580; ^1^H NMR (500 MHz, CDCl_3_) δ 8.73 (s, 1H), 6.72 (d, *J* = 7.9 Hz, 1H), 6.68 (s, 1H), 6.57 (s, 3H), 4.47 (s, 1H), 3.76 (s, 3H), 1.84 (s, 3H). ^13^C NMR (126 MHz, DMSO) δ 158.93, 153.11, 145.59, 143.50, 133.84, 133.50, 119.20, 118.10, 113.51, 109.56, 95.97, 56.30, 53.85, 34.38, 8.18. HRMS (ESI^+^): m/z calculated for [C_15_H_14_N_4_O_3_+H^+^]: 299.1144; found 299.1008

**6-amino-4-(furan-2-yl)-3-methyl-2,4-dihydropyrano[2,3-*c*]pyrazole-5-carbonitrile, 3l:** 91% yield, pale yellow colour. R_f_ = 0.42 (80% EtOAc). M.P 215-217°C. IR (KBr) *υ_max_* (cm^-1^) 3344, 3068, 2912, 1646, 1513, 1498, 1347, 1327, 1243, 1198, 1087, 875, 833, 751, 586; ^1^H NMR (500 MHz, CDCl_3_) δ 7.93 (s, 1H), 7.37 (s, 1H), 6.50 (s, 2H), 6.31 (s, 1H), 6.13 (s, 1H), 4.72 (s, 1H), 2.05 (s, 3H). ^13^C NMR (126 MHz, CDCl_3_) δ 160.10, 154.25, 140.16, 134.33, 119.15, 108.55, 103.95, 93.50, 52.89, 28.59, 8.23. HRMS (ESI+): m/z calculated for [C_12_H_10_N_4_O_2_+H^+^]:243.0882; found 243.0921

**6-amino-4-(thiophen-2-yl)-3-methyl-2,4-dihydropyrano[2,3-*c*]pyrazole-5-carbonitrile, 3m:** 90% yield, pale yellow colour. R_f_ = 0.43 (80% EtOAc/Hexane). M.P 223-224 °C. IR (KBr) *υ_max_* (cm^-1^) 3363, 3083, 2912, 1627, 1599, 1481, 1354, 1300, 1220, 1182, 1066, 867, 833, 761, 612; ^1^H NMR (500 MHz, CDCl_3_) δ 7.93 (s, 1H), 7.22 (d, *J* = 4.3 Hz, 1H), 6.98 (s, 1H), 6.91 (s, 1H), 6.48 (s, 2H), 4.93 (s, 1H), 1.96 (s, 3H). ^13^C NMR (126 MHz, CDCl_3_) δ 159.14, 152.87, 147.90, 134.42, 124.67, 122.92, 122.54, 119.08, 95.80, 56.46, 30.13, 8.28. HRMS (ESI^+^): m/z calculated for [C_12_H_10_N_4_OS+H^+^]:259.0654; found 259.0692.

**6'-amino-3'-methyl-2-oxo-1'H-spiro[indoline-3,4'-pyrano[2,3*-c*]pyrazole]-5'-carbonitrile, 3n:** 92% yield, white solid. R_f_ = 0.50 (80% EtOAc/Hexane). M.P 225-227°C. IR (KBr) *υ_max_* (cm^-1^) 3365, 3093, 2922, 1627, 1701, 1589, 1483, 1344, 1301, 1222, 1181, 1056, 862, 831, 762, 613; ^1^H NMR (500 MHz, CDCl_3_) δ 10.35 (s, 1H), 7.75 (s, 1H), 7.20 (t, *J* = 6.9 Hz, 1H), 7.01 (dd, *J* = 16.7, 6.9 Hz, 2H), 6.91 (d, *J* = 7.6 Hz, 1H), 6.47 (s, 2H), 1.62 (s, 3H). ^13^C NMR (126 MHz, CDCl_3_) δ 183.41, 167.68, 160.45, 146.35, 140.12, 137.61, 133.64, 129.48, 127.45, 123.81, 114.82, 100.32, 61.28, 52.40, 14.22. HRMS (ESI+): m/z calculated for [C_15_H_11_N_5_O_2_+H^+^]: 294.0991; found 294.1021

**6'-amino-1benzyl-3'-methyl-2-oxo-2'H-spiro[indoline-3,4'-pyrano[2,3-*c*]pyrazole]-5-carbonitrile, 3o:** 87% yield, white solid. R_f_ = 0.49 (80% EtOAc/Hexane). M.P 236°C. IR (KBr) *υ_max_* (cm^-1^) 3368, 3098, 2923, 1622, 1716, 1584, 1482, 1343, 1302, 1242, 1161, 1046, 852, 821, 752, 623; ^1^H NMR (500 MHz, CDCl_3_) δ 7.70 (s, *J* = 6.4 Hz, 1H), 7.39 (d, *J* = 7.2 Hz, 2H), 7.34 – 7.30 (m, 2H), 7.27 (d, *J* = 7.3 Hz, 1H), 7.22 (t, *J* = 7.5 Hz, 1H), 7.11 (d, *J* = 7.2 Hz, 1H), 7.07 – 7.03 (m, 1H), 6.84 (d, *J* = 7.7 Hz, 1H), 6.50 (s, 2H), 5.04 (d, *J* = 15.5 Hz, 1H), 4.88 (d, *J* = 15.5 Hz, 1H), 1.46 (s, *J* = 6.3 Hz, 3H). ^13^C NMR (126 MHz, CDCl_3_) δ 175.93, 161.88, 154.56, 140.97, 134.55, 134.25, 130.95, 127.90, 127.64, 126.60, 126.38, 123.64, 122.37, 117.82, 108.12, 94.21, 55.37, 46.20, 42.88, 8.36. HRMS(ESI^+^): m/z calculated for [C_22_H_17_N_5_O_2_+H^+^]: 384.1460; found 384.1506.

**6-amino-5-chloro-3-methyl-2-oxo-2H-spiro[indoline-3,4-pyrano[2,3-*c*]pyrazole]-5-carbonitrile, 3p:** 95% yield, white solid. R_f_ = 0.45 (80% EtOAc/Hexane). M.P 235°C. IR (KBr) *υ_max_* (cm^-1^) 3366, 3092, 2921, 1623, 1715, 1581, 1483, 1342, 1342, 1243, 1162, 1056, 854, 821, 754, 622; ^1^H NMR (500 MHz, CDCl_3_) δ 10.45 (s, 1H), 7.61 (s, 1H), 7.18 (d, *J* = 7.9 Hz, 1H), 7.00 (s, 1H), 6.88 (d, *J* = 8.0 Hz, 1H), 6.33 (s, 2H), 1.69 (s, 3H). ^13^C NMR (126 MHz, CDCl_3_) δ 177.22, 161.85, 157.52, 139.15, 134.41, 133.60, 127.80, 126.50, 123.85, 117.72, 110.23, 93.83, 55.04, 46.81, 8.48. HRMS (ESI+): m/z calculated for [C_15_H_10_N_5_O_2_+H^+^]: 328.0601; found 328.0625

**6'-amino-5-bromo-3'-methyl-2-oxo-2'H-spiro[indoline-3,4'-pyrano[2,3-*c*]pyrazole]-5-carbonitrile,3q:** 93%, white solid. R_f_ = 0.45 (80% EtOAc/Hexane).M.P 240°C. IR (KBr) *υ_max_* (cm^-1^) 3368, 3098, 2923, 1622, 1716, 1584, 1482, 1343, 1302, 1242, 1161, 1046, 852, 821, 752, 623; ^1^H NMR (500 MHz, CDCl_3_) δ 10.79 (s, 1H), 7.49 – 7.20 (m, 4H), 6.90 (s, 1H), 1.60 (s, 3H). ^13^C NMR (126 MHz, DMSO) δ 177.59, 162.49, 155.14, 140.77, 135.11, 134.75, 131.76, 127.31, 118.65, 114.19, 111.72, 94.69, 54.43, 47.51, 9.02. HRMS (ESI+): m/z calculated for [C_15_H_10_BrN_5_O_2_+H^+^]: 372.0096; found 371.9943

**Copies of ^1^H and ^13^C NMR spectra of products:**

**^1^H and ^13^C NMR spectra of products 3a**

**^1^H and ^13^C NMR spectra of products 3b**

**^1^H and ^13^C NMR spectra of products 3c**

**^1^H and ^13^C NMR spectra of products 3d**

**^1^H and ^13^C NMR spectra of products 3e**

**^1^H and ^13^C NMR spectra of products 3f**

**^1^H and ^13^C NMR spectra of products 3g**

**^1^H and ^13^C NMR spectra of products 3h**

**^1^H and ^13^C NMR spectra of products 3i**

**^1^H and ^13^C NMR spectra of products 3j**

**^1^H and ^13^C NMR spectra of products 3k**

**^1^H and ^13^C NMR spectra of products 3l**

**^1^H and ^13^C NMR spectra of products 3m**

**^1^H and ^13^C NMR spectra of products 3n**

**^1^H and ^13^C NMR spectra of products 3o**

**^1^H and ^13^C NMR spectra of products 3p**

**^1^H and ^13^C NMR spectra of products 3q**
